# Term Infant Formulas Influencing Gut Microbiota: An Overview

**DOI:** 10.3390/nu13124200

**Published:** 2021-11-23

**Authors:** Valentina Fabiano, Flavia Indrio, Elvira Verduci, Valeria Calcaterra, Tudor Lucian Pop, Alessandra Mari, Gian Vincenzo Zuccotti, Fugen Cullu Cokugras, Massimo Pettoello-Mantovani, Olivier Goulet

**Affiliations:** 1 Department of Pediatrics, Vittore Buzzi Children’s Hospital, Università degli Studi di Milano, 20154 Milan, Italy; valentina.fabiano@unimi.it (V.F.); elvira.verduci@unimi.it (E.V.); valeria.calcaterra@unipv.it (V.C.); alessandra.mari@unimi.it (A.M.); gianvincenzo.zuccotti@unimi.it (G.V.Z.); 2 Department of Medical and Surgical Sciences, University of Foggia, 71100 Foggia, Italy; 3 European Paediatric Association/Union of National European Paediatric Societies and Associations, 10115 Berlin, Germany; tudor.pop@umfcluj.ro (T.L.P.); fcullu@gmail.com (F.C.C.); mpm@unifg.it (M.P.-M.); 4 Pediatric and Adolescent Unit, Department of Internal Medicine, University of Pavia, 27100 Pavia, Italy; 5 Second Paediatric Clinic, Department of Mother and Child, University of Medicine and Pharmacy Iuliu Hatieganu, 400177 Cluj-Napoca, Romania; 6 Paediatric Gastroenterology, Hepatology and Nutrition, Cerrahpasa Medical Faculty, Istanbul University, Istanbul 34000, Turkey; 7 Department of Pediatrics, Scientific Institute ‘Casa Sollievo della Sofferenza’, University of Foggia, 71122 Foggia, Italy; 8 Association pour l’Activité et la Recherche Scìentifiques, EPA-UNEPSA/ARS, 2000 Neuchâtel, Switzerland; 9 Department of Paediatric Gastroenterology, and Nutrition, Intestinal Failure Rehabilitation Centre, National Reference Centre for Rare Digestive Diseases, Necker-Enfants Malades Hospital, Paris Centre University and Paris-Descartes School of Medicine, 75000 Paris, France; olivier.goulet@aphp.fr

**Keywords:** microbiome modifying formula, probiotics, prebiotics, synbiotics, postbiotics, human milk oligosaccharides, diarrhea, gastrointestinal infections

## Abstract

Intestinal colonization of the neonate is highly dependent on the term of pregnancy, the mode of delivery, the type of feeding [breast feeding or formula feeding]. Postnatal immune maturation is dependent on the intestinal microbiome implementation and composition and type of feeding is a key issue in the human gut development, the diversity of microbiome, and the intestinal function. It is well established that exclusive breastfeeding for 6 months or more has several benefits with respect to formula feeding. The composition of the new generation of infant formulas aims in mimicking HM by reproducing its beneficial effects on intestinal microbiome and on the gut associated immune system (GAIS). Several approaches have been developed currently for designing new infant formulas by the addition of bioactive ingredients such as human milk oligosaccharides (HMOs), probiotics, prebiotics [fructo-oligosaccharides (FOSs) and galacto-oligosaccharides (GOSs)], or by obtaining the so-called post-biotics also known as milk fermentation products. The aim of this article is to guide the practitioner in the understanding of these different types of Microbiota Influencing Formulas by listing and summarizing the main concepts and characteristics of these different models of enriched IFs with bioactive ingredients.

## 1. Introduction

Intestinal colonization takes place immediately after birth [1]. It is highly dependent on the term of pregnancy, the mode of delivery, the type of feeding [breast feeding (BF) or formula feeding (FF)] and the use of antibiotics or proton pump inhibitors [1,2]. Postnatal immune maturation is, as well, highly dependent on the intestinal microbiome implementation and composition [1,2,3]. Type of feeding is a key issue in the human gut development, the diversity of microbiome, and the intestinal function at any age in life [4,5]. By providing bioactive components, human milk (HM) protects the infant against pathogenic infections. It promotes barrier function, stimulates the immune system and facilitates immune tolerance [6,7]. In the past, infant mortality rate in Europe was very high (≥20%) especially in non-breastfed infants [8]. Compared to formula-fed infants, studies of the “intestinal flora” of BF infants showed differences, especially regarding *Bifidobacteria* species [9,10]. Later on, oligosaccharides were identified as the most important bifidogenic factor in HM [10]. Nowadays, it is well established that exclusive BF for 6 months or more, relative to FF, decreases the incidence and/or the severity of a number of infectious diseases [7,11].

As a matter of fact, the composition of the new generation of infant formulas (IFs) aims in mimicking HM by reproducing similar or close beneficial effects on intestinal microbiome and, in turn, on the gut associated immune system (GAIS). Reproducing the beneficial effects of breast milk remains a considerable challenge. Several approaches have been developed currently for designing new IFs by the addition of bioactive ingredients such as HM oligosaccharides (HMOs), probiotics, prebiotics [fructo-oligosaccharides (FOSs) and galacto-oligosaccharides (GOSs)], or by adding the so-called post-biotics also known as milk fermentation products. The aim of this article, the third of a trilogy [12,13], is to assist the practitioner in the understanding of these different types of Microbiota Influencing Formulas (MIFs). Our aim is to help them in the prescription of an IF by listing and summarizing the main concepts and characteristics of these different models of enriched Ifs with bioactive ingredients influencing gut microbiota.

## 2. Methods

This is a literature review of narrative nature [14]. We performed a search in MEDLINE and EMBASE using the search terms infant formula, fermented infant formula, intestinal microbiota influencing formula, HM oligosaccharides (HMOs), prebiotics, probiotics, synbiotics, postbiotics, considering most relevant literature, including clinical trials, review, systematic review and metanalysis published from 2000 onwards, focusing on gastrointestinal clinical and nonclinical outcomes, and excluding allergy, respiratory infections and other non-gastrointestinal outcomes, in line with the topic of the Special Issue.

Authors reviewed relevant literature on a specific topic, with the purpose of identifying what has been accomplished previously, allowing for consolidation, for building on previous work, for avoiding duplication and for identifying omissions or gaps [14], and summarized the content of their paragraph in a short conclusion statement on which there was consent [14].

### 2.1. Probiotics Supplemented IFs

The long-lasting debate on the safety and clinical effects of adding probiotic preparations to IFs, follow-on formulas, and special medical foods has not reached a final consensus and large number of studies are currently ongoing to provide further useful information in this area [15]. During recent years, research has focused particularly on the modifications induced by a probiotic supplementation on infant microbiome and on probiotics’ efficacy and safety on a different number of pathologies [16]. However, IFs are increasingly being supplemented with probiotics and probiotics’ market has expanded globally during recent years [13,16]. Since over two decades the definition of probiotics proposed by the Food and Agriculture Organization of the United Nations and the World Health Organization (FAO/WHO), that describes them as “live microorganisms which when administered in adequate amounts confer a health benefit on the host”, has become the most widely accepted and adopted version worldwide [12,13]. It has proven valuable throughout the years by researchers, legislators, industry and consumers, as it includes a broad range of microbes and applications, while at the same time well it summarizes the fundamental nature of probiotics, characterized for being microbial, viable and beneficial to health [17].

Probiotics are identified by their specific strain, which includes the genus, the species, the subspecies, and an alphanumeric strain designation, as shown in the example in Table 1. The seven core genera of microbial organisms most often used in probiotic commercial products are *Lactobacillus*, *Bifidobacterium*, *Saccharomyces*, *Streptococcus*, *Enterococcus*, *Escherichia*, and *Bacillus* [18].

#### 2.1.1. Rational for the Use of Probiotics in Infant Formula

A rapid colonization of the immature neonatal gut with microbes takes place at birth, further supported by enteral diet and milk [19]. This process starts the organization of a complex ecosystem signals that lead post-natal gut maturation [1,12] towards appropriate digestive and immunological functions [20,21], which further develop through the subsequent and progressive introduction and establishment of different bacteria in infancy and early childhood [22]. Facultative and aerobic bacteria establish first, followed by progressively more strict anaerobes and finally, in adult individuals, the intestinal microbiota includes several hundred, mostly anaerobic, bacterial species [23]. The intestinal microbiota variously contributes to many functions of the gut, which in addition to its primary function of nutrient digestion and absorption, plays an important immunologic role as a protective barrier against the pathogenic microorganisms and the passage of potential harmful macromolecules into the body. Commensal microbes provide the major drive for maturation of the immune system [20]. The bacteria derive from different sources and the colonization pattern, together with several aspects of the gut immune maturation. They are influenced by delivery mode and environmental factors [23] and depend on the interaction with the host-specific gut microbiota. To this regard, early gut colonization takes place through vertical mother-neonate transfer of maternal bacteria, which reach breast milk via an entero-mammary pathway and recent reports emphasized the importance of assessing early host–microbe interactions due to the impact that early gut colonization may have on later health [24]. It is therefore conceivable the potential and important role, played by probiotic supplemented formulas, in influencing and possibly determining the microbiota profile of infant and its positive effect on gut maturation [25].

#### 2.1.2. Microbiota and Gut Development

Gut development is effectively promoted by HM through a direct or indirect activity on gut microbiota [26]. For instance, gut microbiota maturation may be directly influenced by lactoferrin which controls microbes colonization by its anti-microbial function, or indirectly by commensal bacteria [22]. The contribution of other factors may further influence the gut maturation, as in the case of the intake of essential fatty acids and fatty acid desaturase genotype which contribute to the infant immune system maturation [27,28]. Gut microbiota development and gut maturation of formula-fed infants may therefore depend on different functional milk components such as probiotics added to formula. However, still insufficient studies have adequately investigated the relationship between gut microbiota development and gut maturation in early infant life in the same group of subjects, and information on host-microbe crosstalk are currently also still insufficient [22], particularly in reference to probiotic supplemented IFs.

#### 2.1.3. Probiotics Supplemented IFs Tolerability and Safety

The ability to manipulate the composition and metabolic footprints of gut microbiota is longtime well known [12] and IFs supplementation with probiotics has the purpose of modulating the activity of the intestinal microbiota of infants through modifying its balance [29]. Introducing probiotics in formulas in addition to the purpose of conferring health-promoting properties, has an impact on the functional characteristics of the product, including the improvement of taste and other variables known as texture and mouthfeel characteristics/properties, which can make food appealing or not. Probiotic-supplemented IFs do not raise any health concern regarding growth, as stated by the European Society for Pediatric Gastroenterology, Hepatology, and Nutrition (ESPGHAN) [16]. However, few recent cases of *Lactobacillus* or *Bifidobacterium* sepsis in infants receiving probiotics have been reported and there are still many challenges related to the stability and functionality of probiotics in dairy products [29,30]. In neonatology, supplementation with *Bifidobacterium* spp. and *Lactobacillus* spp. is most common. In particular, *B. bifidum* and *L. acidophilus* added to infant formula seem to remain more stable if compared to expressed breast milk and sterile water following storage at 4 °C for 6 h [31].

#### 2.1.4. Probiotics Supplemented IFs in Gastrointestinal Disorders

As reported by a large number of studies [32], the effects on different health conditions elicited by probiotics activities are highly strains specific, although it is somehow difficult to perform an effective comparative analysis of the growing available data, due to continuous changes of probiotics’ taxonomy [30]. A number of recent critical reviews of the literature have evaluated the use, effectiveness and safety of various specific probiotic strains and many different guidelines, position papers and evidence-based recommendations for various clinical indications have been developed in relation to several health conditions affecting children including prevention of nosocomial infections, allergy and gastrointestinal (GI) disorders [16,33,34]. In reference to the latter, various reports have indicated that supplementation of infant formula with probiotics may be useful in certain conditions [34]. A systematic review conducted by ESPGHAN reported the existing evidence related to the safety and health effects of the administration of probiotics supplemented compared with probiotics unsupplemented formula [16]. In general, previous randomized clinical trials (RCTs) report a modest benefit when giving probiotics with the aim to prevent acute gastrointestinal tract infections in healthy infants and children, while meta-analyses and Cochrane reviews have shown that probiotics reduce the number of diarrheal stools and the duration of diarrhea [35,36,37,38,39]. Recent studies have suggested the usefulness of probiotics in preventing infections. Strains of probiotics including *Lactobacillus rhamnosus* GG (LGG), *Streptococcus thermophilus*, *Lactobacillus. casei*, *Bifidobacterium lactis*, or *Lactobacillus reuteri* have been used to supplement infant formula [39], and a reduced incidence rates of gastrointestinal infections were observed in infants receiving formula supplemented with *L. fermentum* [40,41]. Studies on the use of probiotics in the prevention of antibiotic-associated diarrhea have shown that the use of a probiotic-supplemented formula, particularly with *B. lactis* and *S. thermophilus*, reduces the incidence of antibiotic-associated diarrhea [42,43]. The effectiveness of probiotic supplemented formula to reduce colic frequency, crying and irritability in children younger than six months, is debated [44]. For instance, the use of formula with *B. lactis* BL999 and LPR, *L. reuteri* or LGG was not associated to a reduction of these symptoms in children younger than six months, while several studies show a reduced frequency of colic in children older than six months [16,39,45].

#### 2.1.5. Conclusion on Probiotics Supplemented IFs

The increasing worldwide use of commercial probiotic supplemented formulas, and the growth of the related literature have raised the attention of law makers, healthcare professionals and consumers on the advantages and risks related to the use of these products in children and very young infants. The major issues related to the use of probiotics to supplement dietetic products for infants have been well emphasized by many authors. In particular, the ESPGHAN Committee on Nutrition [16] expressed its concern on the important factor of timing, as supplemented formulas are most often initiated early in infancy, if not at birth. Therefore, introducing external factors, such as probiotics, at a time when gut microbiota is developing may influence and permanently affect the development of the ecosystem, leading to currently unclear changes. Finally, the length of administration represents a further element of concern, as the supplemented products may be sometimes administered for prolonged periods, which outcomes have not been sufficiently studied.

Clinical research has shown that probiotic supplementation of newborns and infants with specific bacterial strains through formula is safe. However, specific structured clinical questions supported by well designed, prospective and randomized double-blind studies on adequately selected population will contribute to clarify the health benefits of specific bacterial strains for infants and the usefulness of probiotic supplemented IFs as a mean for their administration (Table 2).

### 2.2. Prebiotics Supplemented IFs

A dietary prebiotic was defined by The International Scientific Association for Probiotics and Prebiotics (ISAPP), in 2010, as “a selectively fermented ingredient that results in specific changes in the composition and/or activity of the gastrointestinal microbiota, thus conferring benefit(s) upon host health”. Later in 2017, ISAPP redefined a prebiotic as “a substrate that is selectively utilized by host microorganisms conferring a health benefit” [46], giving relevance to prebiotics conferring health benefits to the whole body, and not only to the gastrointestinal tract. In the context of increasing interest for the potential health benefits of prebiotics added to food products, they are also frequently presenting IFs with the aim to stimulate the establishment and maintenance of a healthy gut environment, more faithfully resembling that of breastfed infants. The most commonly used and studied prebiotics ingredients include galactooligosaccharides (GOS), polydextrose (PDX), fructooligosaccharides (FOS), 2′-fucosyllactose, lacto-N-neo-tetraose, inulin, oligofructose and galactofructose; with GOS, FOS, and/or PDX, and mixtures of GOS/FOS (the most studied is a 9:1 mixture of short-chain (sc)GOS and long-chain (lc)FOS) being the prebiotics most frequently use and largely studied.

#### 2.2.1. Rational for the Use of Prebiotics in IFs

Contrary to HM, cow’s milk does not contain prebiotic oligosaccharides, that are added to IFs to indirectly act, through selective fermentation that stimulates the growth of *Bifidobacteria* and *Lactobacilli*, for improving intestinal barrier function, protecting against pathogens and enhancing local and systemic immune function [47], with all the health consequences that have been extensively described in the probiotics section.

#### 2.2.2. Prebiotics Supplemented IFs and Intestinal Parameters

Different type of prebiotics substrates may act differently on the growth of intestinal bacteria as, for example, inulin, maltodextrin and PDX have been associated with relatively poor *Lactobacillus* and *Bifidobacterium* growth with respect to GOS and lactulose [48]. A RCT evaluating oligofructose/FOS (50:50, 4 or 8 g/L) or GOS/FOS (8 g/L) supplemented formulas showed that the total number of fecal bacteria increase in all prebiotic groups and more closely resembled that of the breastfed infants [49]. Moreover, other RCTs evaluating GOS/FOS [50,51,52,53] as well as only-GOS enriched formulas [54,55] have demonstrated a selective stimulating effect on the growth of *Bifidobacteria* and/or *Lactobacilli*, resembling the way in which HM acts on microbiome of breastfed infants. Moreover, it has been shown that the beneficial effects on the growth of *Bifidobacteria* and *Lactobacilli* in infants fed with a scGOS/lcFOS (9:1) supplemented formula were maintained even for months after ceasing the prebiotic formula [52]. Studies also showed that FOS-supplemented formulas are associated with increased bifidobacteria [56]; however, one study [57] reported a significant effect only after 1 month of prebiotic-supplemented formula feeding, whereas the effect was no longer significant after 2 months, and another study [58] did not show any statistically significant difference between the infants fed with a prebiotic-enriched formula and those fed an unsupplemented formula. In association with the stimulation of growth of *Bifidobacteria* and *Lactobacilli* [59], some RCTs have also demonstrated that infants fed with prebiotics-supplemented formula (GOS/FOS mixture, PDX and GOS) had shown reduced faecal clostridia [53,54,60]. Similarly, infants fed with a prebiotic-enriched formula (0.4% GOS/lcFOS) showed significantly decreased clostridia percentage in stools after 6 weeks of intervention with respect to infants fed with regular formula (0% vs. 3.29%, respectively); this observation being potentially associated with a reduced risk of intestinal infection [61].

The healthier composition of microbiota in infants fed with prebiotics-added formulas with respect to standard ones may also be associated with other intestinal parameters, such as the faecal pH. The faecal pH has proven to modulate the intestinal environment, and lower faecal pH results in decreased amounts of pathogenic bacteria [62]. Infants fed with a GOS/FOS (90:10, 6 g/L) supplemented formula showed a lower faecal pH after 16 weeks compared to infants fed with a probiotic-enriched formula or with a regular formula [63]; a similar result was also shown in another RCT comparing gastrointestinal parameters, including faecal pH, in infants fed with a GOS/FOS mixture (90:10, 4 g/L) supplemented formula with respect to a standard one [64]. In another study, infants fed with a scGOS/lcFOS-enriched formula showed lower faecal pH after 8 weeks of intervention but not after 26 weeks [53]. The same lower faecal pH was also demonstrated after 4 months of an only-GOS (0.44 g/dl) enriched-formula [54]. The same authors have also demonstrated, in the faeces of infants fed with the GOS-supplemented formula, that the percentage of acid acetic was higher than in the control group; whereas the percentages of propionic and butyric acids were lower, more closely resembling the Short Chain Fatty Acids (SCFAs) pattern that is observed in breastfed infants. SCFAs can improve insulin sensitivity and glucose tolerance, modify lipid metabolism, upregulate the antioxidant glutathione, and affect oxidative stress beneficially in the colon of healthy humans [65]. Recently published review [66] found some differences in microbiota composition and immune parameters in infants fed prebiotic-supplemented formulas compared to those fed standard formulas; however, these findings have been considered inconsistent.

#### 2.2.3. Prebiotics Supplemented IFs and Growth

Growth parameters and other clinical outcomes have been evaluated in various studies in infants being fed with prebiotic-supplemented formulas. A systematic review published in 2011 [67], including 12 RCTs mainly using GOS and mixture of GOS and FOS-enriched formulas, showed significantly increased weight gain in full term infants receiving the prebiotic formula, without any significant impact on length and head circumference. More recently, an updated systematic review [66] showed no significant difference in growth parameters (weight, height and head circumference) in infants fed with GOS/FOS enriched formulas with respect to those fed with standard formulas. A transient increased in body weight was demonstrated at 3 and 6 moths follow-up (*p* < 0.01) in the study by Bruzzese et al. [68] in the group of infants receiving a GOS/FOS-supplemented IF compared to the control group. However, the effect was no longer statistically significant at 9 and 12-months follow-up; head circumference did not show any statistically significant increase in supplemented with respect to unsupplemented group at any time-point. On the contrary, length was significantly increased in the infants receiving the GOS/FOS-enriched IF at all time-intervals (*p* < 0.05). A PDX/GOS-supplemented formula resulted in no differences in anthropometric measures with respect to infants receiving a control formula or a formula supplemented with 0.4 g/100 mL of a prebiotic blend of PDX and GOS from 14 to 60 days of age [60] or from 14 to 120 days of age [69].

#### 2.2.4. Prebiotics Supplemented IFs in Gastrointestinal Disorders

The systematic review by Skorka A, et al. [66] also reported on other clinical outcomes including stool frequency and consistency. In only 4 trials, a higher stool frequency was reported in the supplemented infants with respect to unsupplemented ones. Prebiotics added to IFs included GOS/FOS mixture [70], oligofructose enriched-inulin [71], GOS [54], and PDX/GOS mixture [69]. On the contrary, the previously cited review [66] showed that at least 8 RCTs, published from 2009 onwards, failed to demostrate any significant effects on stool frequency in infants fed with prebiotic-enriched formulas. A number of trials evaluating stool consistency in GOS, FOS/GOS, FOS/GOS/AOS, PDX/GOS and oligofructose-enriched inulin supplemented formulas reported softer stools in infants receiving the prebiotic-supplemented formulas [66], more closely resemblig the stool consistency pattern seen in infants receiving HM.

More specific clinical gastrointestinal (GI) outcomes also include frequency of abdominal pain with crying; several studies found no statistically significant difference between infants fed with a prebiotic-supplemented formula with respect to those fed with a regular formula. A RCT by Vandenplas at al, comparing the GI tolerance of a specific fermented formula (FERM) with scGOS/lcFOS mixture (9:1 ratio and concentration of 0.8 g/100 mL) showed a lower incidence of infantile colic, based on the adapted Roma III criteria, and a lower overall crying time, respectively at 8 and 17 weeks follow-up [72].

The frequency of spitting up/regurgitation, constipation, flatulence, abdominal distention was not significantly different between groups [66,73]. Studies have evaluated different prebiotic supplementations (different ingredients and doses) with treatment durations ranging from 2 weeks to 12 months. Conflicting results were demonstrated for vomiting, with a trial reporting a significantly reduced number of days with vomiting in infants fed with a FOS-enriched IF with respect to infants fed unsuppplemented formula [57], and another showing no difference in either the duration or the frequency of episodes of vomiting in infants receiving the prebiotic formula with respect to those fed with the standard one [74]. Incidence of diarrhea and GI infections was reduced in infants fed with a GOS/FOS-supplemented IF with respect to infants in the control group in the study by Bruzzese et al. [68] and by Ivakhnenko & Nyankovskyy [51], respectively. However, in the already cited systematic review [66] no significant benefits in the reduction of incidence of diarrheal episodes and/or GI infections were found. On the contrary, a RCT showed, at 10 months of age, that the duration of diarrhea was significantly shorter in the group of infants fed with a GOS/FOS enriched formula compared to the control group (*p* = 0.03) [75]. More recently, a double-blind controlled trial showed no difference in the incidence of GI infections in the first year of life in infants fed with a 0.5 g GOS/100 mL supplemented formula compared to those who had received a regular formula [76].

#### 2.2.5. Conclusion on Probiotics Supplemented IF

The ESPGHAN Committee on Nutrition [16] concluded that, due to the limited numbers and heterogeneity of the different included studies, no robust conclusions may be drawn, and prebiotic-supplemented formulas should not be routinely recommended in infants. However, the ESPGHAN Committee on Nutrition has also recognized some potential benefits of prebiotics added to IFs. More already cited studies have shown that adding prebiotics to IFs may be associated with a favorable modulation of microbiome composition and metabolic activity, that promotes the development of an intestinal environment more similar to that of BF infants. These non-clinical effects may be considered in the context of the general action of prebiotics in maintenance of health. Nevertheless, the possible impact of feeding infants with a prebiotic-supplemented IF on most clinical outcomes is still unclear and remains to be more properly clarified in terms of real clinical benefits, potentially durable for infants’ health. However, considering the well-established benefits of HM and the unquestionable recommendation to breastfeed whenever possible, and keeping in mind the central role peadiatricinans play in preventing early harmful events on the infants’ intestinal microbiome [13], prebiotic-enriched formulas, giving their human-milk-mimicking modulation of gut microbiome may be considered as a safe alternative to standard IFs for some, selected, non-breastfed infants, as those with hard stools (Table 3).

### 2.3. HM Oligosaccharides (HMOs) Supplemented IFs

The benefits of HM might be due to bioactive components considered to play a key role in neonatal microbiome implementation and in turn, immune defense and intestinal maturation. HMOs are non-digestible carbohydrates present in high concentrations in HM, existing in a tremendous structural diversity [9,77,78,79]. Over 200 free oligosaccharide structures have been identified from HM [9,77,78,79], which is very much higher than in any mammalian milk [9]. However, only 50 structures are assumed to represent 99% of HMO abundance in HM. The wide variability found in the concentration of HMOs between different women, and even in the same woman, during lactation, is due to polymorphism in the Lewis and Secretor genes [10]. These factors determine both the quantity and pattern of HMOs in milk. Other factors such as maternal age, parity, body weight, body mass index, urban or rural residency, season and lactation may influence the HMOs content of HM [77,80,81,82]. Therefore, HMOs content of HM varies over the course of lactation: 20–25 g/L in colostrum and 10–15 g/L in mature milk. According to the energy content HM (64 kcal/100 mL), HMOs represent 1.5–2.3 g/100 kcal [83]. As a matter of fact, a term infant with a daily consumption of approximately 800 mL of HM would intake approximately 10 g/d of HMOs.

HMOs from HM fall into 3 main categories according to their structure: (i) fucosylated neutral (35–50%); (ii) sialylated acidic (12–14%), and (iii) non-fucosylated neutral (42–55%) [9,78,79]. Today, industry is able to produce oligosaccharides structurally identical to those in HM [82,84]. Some IFs have been enriched with two different HMOs: 20-fucosyllactose (20FL) and lacto-N-neotetraose (LNnT).

Recent and updated reviews have summarized the beneficial effects of HMOs. They are thought to have various mechanisms of action based on the specific structures of these HMOs. As prebiotics, they play a key role in promoting microbiome composition and diversity. They prevent pathogen adhesion and could act as antiviral components and prevention of NEC. HMOs contribute to the maturation of intestinal mucosa and GAIS development. They modulate cell receptor signaling, intestinal barrier functions and production of SCFAs.

#### 2.3.1. Rational for the Use of HMOs in Infant Formula

HMOs are resistant to the gastric acidity and to GI enzymes and reach the colon without being hydrolyzed; just 1% of HMOs is absorbed and join to the systemic circulation. HMOs are “prebiotics” that selectively induce the growth of beneficial (probiotic) organisms such as *Bifidobacterium*, a dominant species in the intestine of breastfed infants [10,85]. *Bifidobacterium longum* subsp. *Bifidobacterium infantis* colonizes efficiently on medium supplemented with HMOs, including 2′-FL, as the sole source of carbohydrate [86,87,88]. *B. infantis* produces short-chain fatty acids (SCFAs), which favor the growth of commensal bacteria instead of pathogenic bacteria [89]. At 3 months of age, infants fed with an IF supplemented with 2′-FL and LNnT are more colonized with beneficial *bifidobacteria* while they experience a decrease colonization with pathogenic bacteria [90]. A study demonstrated that among the 24 probiotic strains, only *Bifidobacterium. longum* subsp., *B. infantis* ATCC 15697 and *B. infantis* M-63 were able to ferment 3′-sialyllactose, 6′-sialyllactose, 2′-FL, and 3′-FL [91]. All these data demonstrate the selective presence of HMO. On the intestinal mucosa, HMOs mimic the glycans, preventing gut epithelial adhesion leading to competition with pathogens (virus bacteria, toxins and/or eukaryotes) and also constituting a biofilm to inhibit the passage of pathogens [92,93,94]. Moreover, they have the ability of being fermented by commensal bacteria (i.e., *Bifidobacteria*) which promote their growth, inhibiting also the colonization by pathogens [95]. A prospective study in infants, demonstrated the beneficial effect of 2′-FL in decreasing in the number of episodes of *C. jejuni*-associated diarrhea [96].

HMOs promote gut maturation increasing growth of *Bifidobacterium*, also inducing the production of SCFAs after the fermentation of *Bifidobacterium* and *Lactobacillus*. SCFAs such as butyrate and propionate can stimulate mucin release, increase mucosal blood flow and modulate intestinal epithelial cell and Goblet cells [97].

HMOs can also directly alter epithelial cell gene expression and the binding ability of certain pathogens to the cell surfaces via changing the expression of cell surface glycocalyx [98,99,100].

#### 2.3.2. HMOs and Immune Modulation

In neonatal period immune system is immature and the balance between TH1/TH2 is not well established, Th2 response is predominant [10,101]. HMOs affect the expression of several cytokines including IL-8, IL-1β, colony-stimulating factor 2 (CSF2), platelet factor 4 (PF4) and IL-17C. They also influence the expression of certain chemokines and cell surface receptors including intercellular adhesion molecule-1 (ICAM-1), intercellular adhesion molecule-2 (ICAM-2), interferon γ receptor 1 (IFNGR1), and IL-10 receptor a (IL10RA) [102]. They modulate the intrinsic expression of cell trafficking-related inflammatory markers, the lymphoid tissue-related signaling pathways and the cytokine and chemokine networks responsible for Th1/Th2 balance [6,93,103]. HMOs may either act locally on cells of the mucosa-associated lymphoid tissues or on a systemic level since 1% of the HMOs are absorbed and reach the systemic circulation [104,105].

Dentritic cells (DCs) are important in the regulation of T cell differentiation and of development of innate and adaptive immune responses during infections and inflammatory diseases. Proper activation of innate immune cells is essential for immune education in early life. Therefore activation of DCs by HMOS is very important for immune development in neonates [102,103]. HMOs are considered to target expression of receptors involved in pathogen recognition, such as toll-like receptors (TLRs), to interact with dendritic cells (DCs) in close proximity to the intestinal epithelial barrier; one sub-population, tolerogenic DC (tDC), which are are functional regulatory T cell (Treg) inducers. tDCs are important for the production of regulatory cytokine (i.e., TGF-β IL-10, IL-27) and reduction of inflammatory cytokine production (i.e., IL-4, IL-12, IL-6, and TNF-α) [103,106].

It was demonstrated that number of interferon-γ-producing CD3+CD4+ and CD3+CD8+ lymphocytes as well as interleukin-13 (IL-13)-producing CD3+CD8+ lymphocytes increases when cord blood T-cells are exposed to acidic HMOs [107]. It was shown also that acidic HMOs also reduce IL-4 production in a subset of lymphocytes and induce IFN-g and IL-10 in human cord blood; indicating that HMOs may downregulate Th2 response in neonatal period and could establish T1/Th2 balance [108].

Goehring et al. demonstrated that the plasma concentration of inflammatory cytokines in the breastfed infants and infants fed with experimental formula supplemented with 2′-FL was markedly lower than that in the infants fed with control formula supplemented with galacto-oligosaccharides [109]. This study indicates that infants fed with a formula supplemented with 2′-FL have lower plasma inflammatory cytokine profile for TNFα, IL1-β, IL1- α and IL 6, which resembles those of a breastfed infant group [109].

#### 2.3.3. HMOs Supplemented IFs, Growth and Gastrointestinal Disorders

Nowadays, some clinical studies involving HMO supplemented IFs are available. As mostly focused on infant growth and tolerance, they showed normal growth and the absence of deleterious effects [90,110]. Marriage et al. studied growth and tolerance of HMO supplemented formula [111]. Formula-fed infants were randomized to 1 of 3 formula with a caloric density of 64.3 kcal/dL. Each formula contained galactooligosaccharides (2.2 g/L or 1.4 g/L), and the 2 experimental formulas contained varying levels (0.2 or 1.0 g/L) of 2′-fucosyllactose (2′FL). The 3 formula groups were compared with a breastfed reference group. There were no significant differences among any groups for weight, length, or head circumference growth during the 4-month study period.

A RCT using HMOs supplemented IFs with 20-FL found immune outcomes similar to that of infants fed HM, while the group receiving an IF supplemented with only GOS showed a different result [109].

A multicenter RCT involved 14 days of age healthy infants fed to 6 months of age: with an IF supplemented with 1.0 g/L 2′fucosyllactose (2′FL) and 0.5 g/L lacto-N-neotetraose (LNnT) as compared to a control IF [90]. It reported the absence of difference in body weight gain, length, head circumference, and BMI as well as in the incidence of GI symptoms, including flatulence, spitting up, and vomiting.

Parschat et al. in a randomized controlled study gave to infants aged ≤ 14 days a mixture of five HMOs (5HMO-Mix) (5.75 g/L total, comprising 52% 2′-fucosyllactose, 13% 3-fucosyllactose, 26% lacto-N-tetraose, 4% 30 -sialyllactose, and 5% 60 -sialyllactose) or an IF without HMOs for 4 months, with the others exclusively breastfed [112]. There were no differences in weight, length, or head circumference gain between the two formula groups. The 5HMO-Mix was well tolerated, with 5HMO-Mix and breastfed infants producing softer stools at a higher stool frequency than the control IF group. A study compared the intestinal microbiome of infants fed HMOs supplemented IF [20FL (1 g/L) and LNnT (0.5 g/L)] and those fed without milk. The HMOs group had intestinal microbiome more similar to those breastfed at 3 months of age, with *Bifidobacterium* being more abundant, while *Escherichia coli* and *Peptostreptococcaceae* were less abundant. Fecal concentrations of SCFAs in infants fed the HMOs supplemented formula, were more similar to those in breastfed infants [87,89].

#### 2.3.4. Conclusion on HMOs Supplemented IFs

According to these clinical data, IF enriched with 2 HMOs, 2o FL and LNnT, are considered as safe by the European Union as well as by the US and approved for use as food [113]. They are already available in several countries. However, the number of RCTs that evaluated the effect of these HMOs supplemented IFs on infant health are scarce, generating relatively limited evidence of the potential preventive effects of supplemented IFs with one or both of these HMOs. Therefore, more controlled clinical trials are needed for promoting routine supplementation of IFs. (Table 4)

### 2.4. Synbiotics Supplemented IFs

As knowledge about microbiota evolves, new therapies arise with the aim of preventing the dysfunction or restoring homeostasis. Synbiotics supplementation is an evolving and advancing field which was initially thought as a mix of prebiotics and probiotics [65]. This was actually inconclusive as there were no indications on which should be the interaction between the synbiotic and the microbiota. Indeed, a recent consensus panel by ISAPP gave a newer, more specific definition: “a mixture, comprising live microorganisms and substrate(s) selectively utilized by host microorganisms, that confers a health benefit on the host”. In fact, this statement was made more inclusive by creating subcategories: “synergistic”, where the substrates do not need to be prebiotics but to be metabolized only by the co-administered microorganism with a synergistic beneficial effect on the host; “complementary”, where each of the component must fulfill the requirements for prebiotic or probiotic [114].

#### 2.4.1. Rational for the Use of Synbiotics in Infant Formula

In the past 20 years, synbiotics have been studied to achieve a formula milk that resembles the mothers’ one, in order to recreate a gut microbiota that is similar to breastfed infants. Synbiotics should be more beneficial than prebiotics or probiotics alone due to synergistic effects. As a combination, these mixtures might offer an added effect on the microbiota homeostasis. Mechanisms of action include resistance to colonization by pathogens through blockage of adhesion sites, production of inhibitory substances, degradation of toxin receptors, stimulation of immunity, and competition for nutrients [115]. *Lactobacilli* and *Bifidobacteria* are commonly used in these synbiotics because, generally, they can adhere to the intestinal wall and produce nutrients such as butyrate, hinder the adhesion and inhibit the growth of pathogens, as well as stimulating the immune system and enrich the normal flora [116]. Prebiotics enhance *Bifidobacteria* and *Lactobacilli’s* survival and proliferation. Moreover, they have been shown to dampen the growth of pathogenic genera such as *Clostridium* [117]. It is now recognized that prebiotics in the synbiotic mixture enhance the survival of probiotic bacteria and stimulate the host’s endogenous bacteria [118]. However, the superiority of synbiotics over probiotics or prebiotics has not been well established.

#### 2.4.2. Synbiotics Supplemented IFs and Growth

Consumption of synbiotic-enriched IF has increased in the past decades, although, to date, clinical data regarding their use are limited. Puccio et al. [119] conducted one of the first studies to evaluate the tolerability and safety of combined administration of probiotics and prebiotics in formula milk. In this trial, 138 non-breastfed infants after day 14 of life were enrolled to receive either an experimental formula containing 2 × 10^7^ colony forming unit (CFU) of *Bifidobacterium longum* BL999 and a mixture of prebiotics (90% GOS and 10% FOS) or a standard infant formula until 112 days of age. The investigators demonstrated equivalent weight gain between the two groups, whereas no significant difference was found in length, head circumference, or incidence of adverse events between the two groups. Infants in the intervention group had a lower incidence of constipation (*p* = 0.03) and significantly higher stool frequency (2.2 ± 0.7 versus 1.8 ± 0.9 occurrences/day, *p* = 0.018) underscoring better tolerability of the formula.

One of the first trials on the long-term safety of synbiotic-containing formulas was conducted in Finland by Kukkonen et al. [120]. It included 925 mothers with children at high risk for allergy were randomized to receive a mixture of *Lactobacillus rhamnosus* GG and LC705, *Bifidobacterium breve* Bb99, and *Propionibacterium freudenreichii* subsp. *shermanii* JS at a dose of 8–9 × 10^9^ CFU, or a placebo, twice daily for 4 weeks before delivery. Infants received the same probiotics and 0.8 g of GOS or a placebo, daily, for 6 months after birth. At the end of the 2-year follow-up, no difference was detected in the growth of infants in the two groups, neonatal morbidity, and functional disorders such as infantile colic.

In another trial [121] including 284 infants randomized to receive, from 2 to 16 weeks of age, a control formula or one of the following 3 different study formulas containing the first *Bifidobacterium longum* BL999 (BL999), *Lactobacillus rhamnosus* LPR (LPR), the second BL999, LPR and 4 g/L of 90% GOS/10% short-chain-FOS, and the third BL999, *Lactobacillus paracasei* ST11 (ST11) and 4 g/L GOS/short-chain-FOS. Weight gain was demonstrated in all groups; in contrast, there were no significant differences between study groups in recumbent length, head circumference, digestive tolerance, and frequency of adverse events. Vlieger et al. [122] conducted another RCT randomizing a total of 126 infants into 2 groups: the first received a formula containing *Lactobacillus paracasei* subsp. *paracasei*, *Bifidobacterium animalis* subsp. *lactis* and GOS (0.24 g/100 mL) (synbiotic group); the second received the same prebiotic-containing formula without probiotics (prebiotic only group). The duration of the intervention was 3 months. No significant difference was observed for gain in weight, length, and head circumference between the two groups. In an additional RCT, 146 infants were randomized to receive for 6 months either a formula enriched with GOS and FOS, or another formula with synbiotics (FOS/GOS and *Lactobacillus paracasei* subsp. *paracasei* strain F19 at a dose of 10(9) CFU) [15]. In both groups, growth parameters were similar. In a more recent trial conducted on healthy term infants a 30% fermented infant formula (FIF) (using *Bifidobacterium breve* C50 and *Streptococcus thermophilus* 065) with a specific prebiotic mixture (short-chain GOS and long-chain FOS (9:1, 0.8 g/L)) was compared to a standard formula and a group of breastfed infants [73]. Again, the experimental formula was well tolerated, daily weight gain and growth outcomes were equivalent and close to those of breastfed infants. A tendency in increased stool frequency has been also observed.

A recent meta-analysis concluded that the use of synbiotics have not any significant effect on growth parameters [123].

#### 2.4.3. Synbiotics Supplemented IFs and Intestinal Parameters

In a multicenter RCT conducted in South Africa, the effect of a formula supplemented with *Bifidobacterium animalis* subsp. *lactis* strain CNCM I-3446 (10(7) CFU)/g and a mixture of bovine milk-derived oligosaccharides (BMOS) generated from permeated whey (containing GOS and milk oligosaccharides such as 3′- and 6′-sialylactose) on intestinal *Bifidobacteria* levels of infants born to human immunodeficiency virus (HIV)-positive mothers has been studied [124]. A total of 421 infants were randomized into 4 parallel groups: the first two groups were infants born by cesarean section (CS) assigned to the study formula (*n* = 92) or a control formula (*n* = 102); the other two groups consisted of infants born vaginally randomized to the study (*n* = 115) or control (*n* = 113) fomula. The intervention period was 6 months. The tested formula induced a strong bifidogenic effect in both modes of delivery compared with the control formula, succeeding in correcting the low level of *Bifidobacteria* found in infants born by CS. Faecal pH was significantly lower in infants fed with the tested formula compared with control at 10 days and 4 weeks regardless of the type of delivery, while, at 3 months, this acidification effect persisted only among infants born by CS.

The same bifidogenic effect was also observed in a trial involving an IF supplemented with a mixture of GOS and FOS [50] but also in another trial using synbiotics-enriched infant formula [125]. In particular, the latter is a trial conducted in Germany in healthy infants randomized to receive an extensively hydrolysed formula with the prebiotic short-chain-FOS/long-chain-FOS (9:1) mixture with *Bifidobacterium breve* M-16V (*n* = 45) or the same formula without this synbiotic (*n* = 57) for a 13-week intervention period [125]. A statistically significant higher percentage of fecal *Bifidobacteria* was found at 13 weeks in the synbiotic group compared to the control group (60% vs. 48%, *p* = 0.014). In addition, a lower level of potential pathogens such as *Clostridium lituseburense/Clostridium histolyticum* was observed in the synbiotic group compared with the control group at both baseline and at the end of the intervention time (*p* = 0.003 and *p* = 0.013, respectively).

The doses of probiotics and synbiotics used in the various trials in infants and children range from 10(8) to 10(11) CFU/day [126]; on the other hand, in HM the number of bacteria is quite lower (10(3) to 10(5) CFU/mL, about 10(6)–10(8) CFU/day) [127]. Therefore, it is critical to identify the effects of different doses of synbiotics on the infant’s gut microbiota.

In a recent trial conducted by Phavichitr et al. a synbiotic mixture (0.8 g/100 mL short-chain GOS/long-chain FOS (9: 1) and *Bifidobacterium breve* M-16V at 10(4) CFU/mL or 10(6) CFU/mL) similar in doses to that found in HM on 290 healthy infants aged 6 to 19 weeks [128]. After 6 weeks of intervention, a significant increase in the proportions of *Bifidobacteria* and a reduction in the abundance of *Clostridium difficile* have been observed. Even though a lot of studies showed the role of synbiotic in modifying gut microbiota composition, a lack of well-designed studies does not allow final conclusions.

#### 2.4.4. Synbiotics Supplemented IFs in Gastrointestinal Disorders

Stool pattern (frequency and consistency) have been evaluated in infants receiving synbiotic-enriched IF. In the trial by Chouraqui et al. stool frequency was significantly higher in infants who received formula containing BL999, LPR, and GOS/short-chain-FOS compared with the control group (2.1 vs. 1.6 per day, *p* = 0.03) [121]. Similarly, in another trial, infants in the synbiotic group had a higher frequency of stools during the first 3 months than the prebiotic-only group (1.52 vs. 1.29 times/day, respectively; *p* = 0.04) and the stools had a greater consistency (2.57 vs. 2.36, respectively, *p* = 0.05) [122]. No significant differences between groups were observed in crying and sleeping hours, antibiotic use, number of parent-diagnosed infections, number of adverse events and visits to the general practitioner [122]. In the study by Meli et al., infants fed BMOS-containing formula had more frequent (*p* < 0.0001) and less hard stools (*p* = 0.0003) also in the study by Meli et al. [129]. A higher stool frequency was reported in the groups supplemented with BMOS and this effect is similar to that described in previous studies on oligosaccharides added to the formula [119] and to that seen in breastfed infants. The partially fermented formulas with prebiotics made stool consistency lower than that of those fed the control formula and more similar to that of breastfed infants [73,130].

Among the beneficial effects of synbiotic-enriched formulas, it has also been hypothesized that these formulas could reduce the incidence of infectious diseases [68,118,131]. Based on the assumption that infectious diseases are a major public health issue, it is likely that synbiotic IFs could be able to mimic the preventive and beneficial effects of maternal breastfeeding on infectious diseases.

In the londitudinal study by Picaud et al. [132] infants fed with a follow-on IF enriched with FOS (28 mg/g of powder) and two probiotic strains (*Bifidobacterium longum* at 10(7) CFU/g of powder and *Streptococcus thermophilus* at 10(6) CFU/g of powder) for three months had less infectious diseases than infants fed standard formula (31. 0% vs. 40.6%; *p* = 0.005) and, specifically, significantly less GI infectious diseases (3.5% vs. 6.8%; *p* = 0.03). Similar results were also demonstrated in another Spanish RCT conducted in children 1–6 months of age fed a formula supplemented with *Lactobacillus fermentum* CECT5716 at a concentration dose of 10(7) CFU/g of formula and GOS (0.3 g/100 mL) or a control formula containing only the same concentration of GOS for 5 months [40]. In this study, the incidence rate of GI infections in infants in the synbiotic formula-fed group was 3 times lower than in the control group (*p* = 0.018). This is the same result obtained in another study in which a reduction of 46% in the incidence of GI infections using the same strain of *Lactobacillus fermentum* was observed [41], and in other RCTs in which other synbiotic formulas were effective in preventing community-acquired GI infections and diarrhea episodes [41,132,133].

In the already cited RCT conducted by Meli et al., there were no statistically significant differences in the frequency of flatulence, fussing, vomiting, crying and spitting up. On the contrary, a higher incidence of colic in the BMOS formula group was observed compared to the control group and the authors hypothesized that it could be due to a higher level of oligosaccharides added to the formula, compared to the levels used previously [119,134]. However, the risk of colic was not significantly different between the control group and the group fed formula with BMOS and probiotics, which suggests the hypothesis that the addition of probiotics may favorably modulate the risk of colic attributable to oligosaccharides.

#### 2.4.5. Conclusion on Synbiotics Supplemented IFs

At present, there are too limited data available in the literature on IFs supplemented with synbiotics to provide specific therapeutic indications. Back in 2011, in the systematic review on the use of synbiotics of the ESPGHAN Committee on Nutrition [11] caution was required in their use given the paucity of data, although no adverse effects were reported and their use was considered as safe.

IFs enriched with synbiotics have shown positive effects on modulation of microbiome composition and metabolic activity, leading to a beneficial impact on gut immune functioning, GI symptoms and creating an intestinal environment that is more similar to that of breastfed infants. In a scientific world increasingly oriented towards the search for personalized target therapies, further investigations should be encouraged to evaluate the possible impact of synbiotic-enriched IF on gut microbiota, growth and infectious diseases. This is a large field for more well-designed long term clinical trials needed to establish which types of prebiotic and probiotic species and strains, single-strain, or multi-strain, the optimal doses, the duration of intake and of course the safety of synbiotics. For these reasons, to date, there are no recommendations on the routine use of formula supplemented with synbiotics in infants. (Table 5)

### 2.5. Postbiotics Supplemented IFs

In the last 20 years, different terms referring to inactivated or killed microorganisms as paraprobiotics, non-viable probiotics, heat-killed probiotics, tyndallized probiotics [135] have been used. In 2019 The ISAPP consensus statement [135] defined postbiotics as “preparation of inanimate microorganisms and/or their components that confers a health benefit on the host”. The adjective ‘inanimate’ has been specifically chosen to refer to microorganisms that are no longer viable but still retain their functions.

The currently defined classes of postbiotics [136], including both metabolites and fragments of microorganisms that may exert a beneficial effect in the host include cell-free supernatants (derived from *L. rhamnosus* GG, *L. acidophilus*, *L. casei*, *L. plantarum* and from yeast: *S. cervisiae*, *S. boulardii*), exopolysaccharides (derived from *L. plantarum*. *L. helveticus*, *L. kefiranofaciens*, *β-glucans*), antioxidant enzymes such as glutathione peroxidase, peroxide dismutase, catalase, NADH-oxidase (derived from *L. fermentum*, *L. plantarum*, *L. delbruekii* subsp. *lactis*), cell wall fragments as bacterial lipoteichoic acid (derived from *Lactobacillus* and *Bifidobacteria*), SCFAs: acetic, propionic and butyric acids (from the fermentation of plant polysaccharides in gut microbiota), bacterial lysates (obtained by chemical or mechanical degradation of Gram-positive and Gram-negative bacteria), postbiotics derived from dietary polyphenols (urolithin A, equol and 8-prenylnarigenin). They have a heterogeneous composition and there are several techniques (chemical or mechanical) by which postbiotics can be obtained.

#### 2.5.1. Rational for the Use of Postbiotics in Infant Formula

There are some specific indications about the formulation of postbiotics as reported in ISAPP Consensus Statement [135]: the progenitor microorganism must be characterized at the molecular level to identify the corresponding product and trace of any potentially ‘unsafe’ genes, must provide a detailed description of the inactivation process and matrix, must confirm successful inactivation, must demonstrate the ability to bring benefit with quality clinical studies, must provide a detailed description of preparation’s composition and assessment of safety in the final host and context of use.

#### 2.5.2. Postbiotic, Microbiota and Metabolic Activity

Data collected until now can partially explain the complex effects of postbiotics, but it seems that they can act with pleiotropic properties on gut epithelium and microbiota, immune system, systemic metabolism, and the nervous system [135]. Although the effect may be temporary, molecules such as lactic acid and bacteriocins (still present despite inactivation) may have direct antimicrobial activity. They may also act indirectly by modulating intracellular cross talk (quorum sensing) or by providing valuable substrates for certain strains by supporting their proliferation. Postbiotics can then compete with resident microorganisms for adhesion sites in the presence of fimbriae or lectins [135].

SCFAs in postbiotic preparations have been shown to influence the function of the intestinal barrier by acting on tight junctions and, also, to protect it from the negative action of lipopolysaccharides if present at sufficient levels. Moreover, some proteins (Msp1/p75 and Msp1/p40 or HM0539) enhance epithelial barrier function and exopolysaccharides can reduce the inflammation promoting barrier function [135].

Numerous bacterial interaction structures can stimulate an immune response. Among these, peptidoglycans and derivatives have been shown to interact with NOD2, lipoteichoic acid with Tool Like Receptor 2 (TLR2) and TLR6, lipopolysaccharides from certain Gram- (E. coli Nissle for example) with TLR4, beta-glucans and lipoproteins with TLR2. Other immunomodulatory metabolites such as histamine, SCFAs or branched-chain fatty acids have shown a role in various immune responses, such as suppressing Nf-KB. Metabolites as lactic acid may mediate immune effects through GPR31-dependent dendrite protrusion of intestinal CX3CR1+ cells [135,136].

As with the microbiota, the effects on the metabolism of metabolites or enzymes expressed by postbiotics can be direct or indirect. Succinate, for example, a substrate of intestinal gluconeogenesis, improves glycaemic control in vivo; acetate, on the other hand, has been shown to regulate appetite centrally. SCFAs can improve insulin sensitivity and glucose tolerance, modify lipid metabolism, upregulate the antioxidant glutathione, and affect oxidative stress beneficially in the colon of healthy humans [135].

Microorganisms can produce various neuroactive compounds (serotonin, dopamine, acetylcholine and GABA) that can act on both the enteric and central nervous systems with the potential to modulate behavior and cognitive function and others that bind receptors expressed in the brain (indoles and bile acids). Microbial enzymes can also metabolize dietary precursors for host neurotransmitter synthesis, such as tryptophan (for serotonin) and tyrosine (for dopamine) [135]. In addition, microbial metabolites, such as SCFAs, if present in a sufficient quantity in the postbiotic preparation, could stimulate enterochromaffin cells to produce serotonin, which can subsequently enter the bloodstream. In addition, it appears that serotonin can also be produced by enterochromaffin cells, which in turn are stimulated by SCFAs if present in sufficient quantity in the postbiotic. In clinical studies, SCFAs have also been shown to play a role in eating habits by stimulating the release of anorexigenic hormones, such as peptide 1 and peptide YY, to promote satiety.

**Figure 1:**
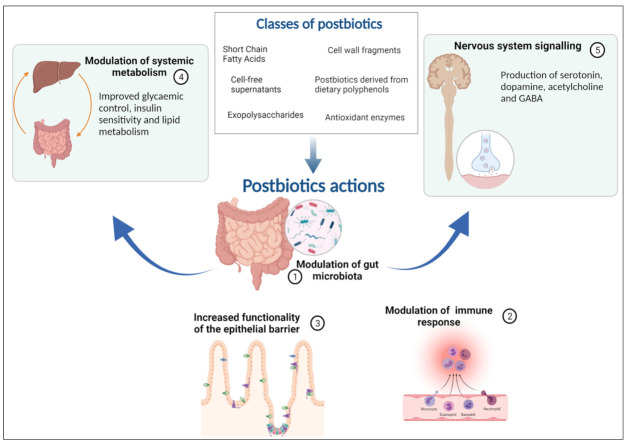
The five mechanisms of action of postbiotics: modulation of the resident microbiota, enhancement of epithelial barrier functions, modulation of local and systemic immune responses, modulation of systemic metabolic responses, and systemic signalling via the nervous system.

Finally, although some B vitamins may also be present in postbiotics and have an important role in central nervous system function, it is still unclear how much of these substances are present in postbiotics [135] (Figure 1).

#### 2.5.3. Postbiotics Supplemented IFs in Gastrointestinal Disorders

Most of the RCTs on postbiotics studied the benefits of FIF compared to breast milk or standard formula and the clinical use in GI and allergic diseases. Among the most investigated are the postbiotics derived from *Bifidobacterium breve* C50, *Streptococcus thermophilus* 065, *Lactobacillus acidophilus* LB, *Lactobacillus paracasei* CBA L74 or 33.

In healthy infants, a meta-analysis [137] based on studies from Italy and France showed that fermented infant formula (FIF) *Bifidobacterium breve* C50 and *Streptococcus thermophilus* 065 do not offer clear additional benefits compared to standard IF, although there are GI benefits that cannot be excluded and no adverse effects [138,139,140,141,142,143,144].

FIF using *Bifidobacterium breve* C50 and *Streptococcus thermophilus* 065 combined with prebiotics (scGOS/lcFOS) are safe to use and well-tolerated [72,73,145]. Studies performed in healthy term infants proved that there are effects in colics, reducing overall crying time [72], with no difference on weight gain [130,145] and softer stool consistency than standard formula and closer microbiome composition and metabolic activity towards breastfed infants [73,130].

Also, the thymus index (as a marker of the immune competence level) was similar with breastfed infants and effects were comparable to those of the bacteria composing the intestinal microbiota [138]. After its use, higher bifidobacterial levels and *Bifidobacterium longum*/*Bifidobacterium infantis* ratio compared to standard formula were proved [140]. Using FIF with heat-inactivated *Bifidobacterium breve* C50 and *Streptococcus thermophilus* 065 in premature infants during the hospital stay for 2–5 weeks showed good clinical tolerance, lower abdominal distension, benefits in inflammatory markers (lower faecal calprotectin), no significant changes in bacterial colonization [146]. In a previous prospective study in healthy neonates, after a 3-month intervention, there was no difference in calprotectin level [147]. Heat-killed *Lactobacillus acidophilus* LB was used in infants or children with acute diarrhea [148,149,150] with a reduction of the duration of diarrhea in hospital, in non-rotaviral infection by one day [148], but not in outpatient [151]. The same reduction of the severity of episodes with fewer cases of dehydration, fewer medical consultations, fewer ORS prescriptions, fewer switchers to another formula (milder course of the disease) was also proved for heat-inactivated FIF with *Bifidobacterium breve* C50 and *Streptococcus thermophilus* 065 in healthy term infants [141]. Contrary to these results, a double-blind RCT from Pakistan, including healthy infants with high risk for diarrhea-related mortality, demonstrated that heat-inactivated *Lactobacillus acidophilus* had no difference in diarrhea prevalence compared to placebo [152]. Another randomized study showed that in healthy infants under 5 months of age, the use of heat-inactivated *Bifidobacterium breve* C50 and *Streptococcus thermophilus* 065 had a good rate of acceptance, the infants presenting less diarrhea [153]. Three RCTs analyzed the adverse effects of postbiotics in infants and children and concluded that there was no significant difference between study and control groups [149,154,155] and many other studies reached the same conclusion [72,138,139,140,141,142,145,156].

#### 2.5.4. Conclusion on Postbiotics Supplemented IFs

The use of postbiotics seems to bring a benefit for healthy term neonates in developing the microbiota or immunomodulation when used in functional foods. There is limited evidence to recommend using postbiotics in acute gastroenteritis. Studies showed that postbiotics are well tolerated and have no adverse effects in infants and children. As postbiotic signatures are dependent on bacterial strains and processes, the safety and suitability of specific postbiotics in infant formula remains to be confirmed. Also, future studies should be realized to establish the recommended dosage and their effects in children (Table 6).

### 2.6. MIFs and Allergy

Although the present review is aimed to consider main concepts and characteristics of MIFs specifically focusing on gastrointestinal outcomes, IFs enriched with different bioactive ingredients, including probiotics, prebiotics, HMOs, postbiotics and synbiotics, have also been studied in allergic infants. In fact, a healthy intestinal microbiota plays an important immunologic role and an altered patterns of early gut colonization may be associated with increased risk of developing allergic diseases, particularly food sensitization, and especially cow’s milk allergy (CMA), and atopic eczema.

HMOs promote gut maturation, increasing growth of Bifidobacterium, inducing, also, the production of SCFAs after the fermentation of Bifidobacterium and Lactobacillus. SCFAs such as butyrate and propionate can stimulate mucin release, increase mucosal blood flow and modulate intestinal epithelial cells and Goblet cells [97]. The maturation of the intestinal barrier function is very important during the neonatal period as the first line of defense, this barrier being also important for the prevention of allergy. Moreover, proper activation of innate immune cells is essential for immune education in early life, and the capability of HMOs to activate dendritic cells (DCs), deeply involved in the regulation of T cell differentiation and in development of innate and adaptive immune responses, is very important for immune development in neonates [103].

Probiotics and prebiotics may modulate immune development throughout several different pathways, thus their role in allergy prevention and treatment have been extensively studied. The World Allergy Organization (WAO), in 2015, has recommended the use of probiotics in pregnant and lactating women and in non-exclusively breastfed infants at high risk of allergic disease [157]; nevertheless, recommendations of both probiotics and prebiotics were based on very low quality evidence. The WAO guideline panel, based on the Grading of Recommendations Assessment, Development and Evaluation (GRADE) in 2016, suggested the use of prebiotic supplementation in infants who were not exclusively breastfed. Again, recommendations were based on a very low certainty of evidence [158].

Conversely, the Academy of Allergy and Clinical Immunology [159] and ESPGHAN [16] did not recommend the use of probiotics and/or prebiotics for the prevention of allergic diseases. Beneficial effect of prebiotics in allergy, and specifically in CMA, is still inconclusive. Whey-based extensively hydrolyzed formula (EHF) containing two HMOs was tolerated and could be recommended to CMA patients [160]. In a recent meta-analysis considering studies comparing the use of amino acid-based formulas containing synbiotics in infants with CMA, versus amino acid-based control formulas, both formulas were shown to be effective in managing allergic symptoms [161].

However, considering that the maturation of the infant immune system occurs mainly in the first months of life, that are the ideal time for prevention of the development of allergic diseases [162] it can be assumed that the use of synbiotics IFs might be more effective in inducing tolerance as, individually, both prebiotics and probiotics have immunomodulatory effects. Postbiotics have also been studied with contrasting results, with studies showing a decrease in the positive skin-prick test responses, without any change in proportion of children with CMA [139] and others indicating that liveable, but not heat-inactivated bacteria may be beneficial on infants affected by CMA [163]. The use of bacterial lysates to influence the immune system was analyzed regarding allergic diseases; and published meta-analyses and systematic reviews showed a reduction of the incidence of allergic rhinitis episodes [164] and symptoms of atopic dermatitis [165].

Strong evidence is missing for recommending MIFs in the prevention of allergic diseases; however, bioactive ingredients, by balancing intestinal bacterial environment, favoring immune maturation, stimulating SCFAs production and enhancing intestinal barrier may favorably act in the modulation of those pathogenic mechanisms that have been associated with allergy development.

## 3. Conclusions

Type of feeding is a key issue in the human gut development, the diversity of microbiome, and the intestinal function at any age in life. Whereas breastfeeding is the reference, the increasing efforts industry is making in the production of IFs that may qualitatively resemble and act as close as possible to HM have led to the supplementation of different bioactive ingredients, including probiotics, prebiotics, synbiotics, postbiotics and HMOs. Concomitantly, scientific data on the benefits of MIF is continuously growing, with much evidence indicating overall positive effects on microbiome composition and metabolic activity. Some benefits are also emerging from RCTs evaluating the clinical impacts these enriched formulas may have on the health of FF infants. Nowadays, none of these IFs has demonstrated conclusive superiority, while clear evidence still lacks. For supporting the challenge of mimicking the benefits of breast milk, more RCTs are still needed. Their aims are to better clarify, if present, which benefits the supplementation of IFs with MIF may practically have on different clinical aspects, including prevention of GI disorders and infections, and their durability over time. So far, whereas no routine recommendations can be done, MIF have generally proven to be well-tolerated and safe in ensuring infants’ normal growth.

## Figures and Tables

**Table 1:** Example of nomenclature used to identify commercial strains of probiotics.

Genus	Species	Subspecies	Strain Designation	Strain Abbreviation
*Lactobacillus*	*rhamnosus*	*NA*	GG	LGG
*Bifidobacterium*	*animalis*	lactis	DN-173010	*Bifidus regularis*
*Bifidobacterium*	*longum*	longum	36624	*Bifantis*

**Table 2:** Summary of beneficial effect of MIFs enriched with Probiotics.

MIFs Enriched with Probiotics—Key Points
• The intestinal microbiota (IM) contributes to the early and healthy development of gut functions
• Commensal microbes are essential for maturation of the immune system
• IFs supplementation with probiotics has the purpose to modulate the activity of the intestinal microbiota of infants by modifying its balance
• Probiotics enriched IFs have modest benefit in preventing acute gastrointestinal tract infections in healthy infants
• Probiotics reduce the incidence of antibiotic-associated diarrhea
• The effectiveness of probiotic supplemented IFs to reduce colic frequency, crying and irritability is debated
• Major issues related to the use of probiotics: timing, duration of treatment

**Table 3:** Summary of beneficial effect of MIFs enriched with Prebiotics.

MIFs Enriched with Prebiotics—Key Points
• Prebiotics stimulate the establishment and maintenance of a healthy gut environment
• Commensal microbes are essential for maturation of the immune system
• Prebiotics act through selective fermentation in the GI tract, which stimulates the growth of bifidobacteria and *Lactobacilli*
• Different types of prebiotics substrates act differently on the growth of intestinal bacteria
• Prebiotics enriched IFs are associated with lower intestinal pH, with a SCFAs pattern more similar to breastfed infants
• Prebiotics enriched IFs are not associated with increased frequency of stool
• Prebiotic supplemented formulas may be considered in infants with hard stool

**Table 4:** Summary of beneficial effect of MIFs enriched with HMOs.

MIFs Enriched with HMOs—Key Points
• HMOs are non-digestible carbohydrates present in high concentrations in human milk
• HMOs play a key role in promoting intestinal microbiome composition and diversity
• HMOs prevent pathogen adhesion and could act as antiviral components
• HMOs-enriched IFs result from the addition of industrially produced HMOs of two types 2o FL and LNnT
• HMOs-enriched IFs are associated with normal infants’ growth
• Incidence of GI symptoms, including flatulence, spitting up, and vomiting did not differ between HMOs-supplemented and unsupplemented IFs
• IF enriched with 2 HMOs, 2o FL and LNnT, are considered as safe and approved for use as food
• There is limited evidence regarding the potential preventive effects of supplemented IFs with one or both the above-mentioned HMOs

**Table 5:** Summary of beneficial effect of MIFs enriched with Synbiotic.

MIFs Enriched with Synbiotics—Key Points
• The substrates do not need to be prebiotics but should be metabolized only by the co-administered microorganism with a synergistic beneficial effect on the host
• Each component must fulfill the requirements for prebiotic or probiotic
• Synbiotics might offer an added effect on the intestinal microbiota homeostasis
• Infants’ growth parameters did not differ between synbotics-supplemented and unsupplemented IFs
• Synbiotics enriched IFs seem to be associated with reduced incidence of GI infections
• Frequency of flatulence, fussing, vomiting, crying and spitting up is not reduced in infants fed with synbiotics-enriched IFs
• No specific therapeutic indications may be provided for synbiotics-enriched IFs

**Table 6:** Summary of beneficial effect of MIFs enriched with Postbiotic.

MIFs Enriched with Postbiotics—Key Points
• Postbiotics are metabolites and fragments of microorganisms resulting from fermentation with live bacteria
• Postbiotics may exert a beneficial effect in the host by pleiotropic properties
• Postbiotics influence gut epithelium and microbiota, immune system, systemic metabolism, and the nervous system. Synbiotics might offer added beneficial effects on intestinal microbiota homeostasis
• Infants’ growth parameters did not differ between postbiotics-supplemented and unsupplemented IFs
• Postbiotics-enriched IFs are associated with softer stool
• Contrasting results on the efficacy of postbiotics in reducing diarrhea episodes: there is limited evidence to recommend using postbiotics for prevention or treatment of acute gastroenteritis

## Data Availability

Not applicable.
